# Enhanced macrophage tropism of HIV in brain and lymphoid tissues is associated with sensitivity to the broadly neutralizing CD4 binding site antibody b12

**DOI:** 10.1186/1742-4690-6-69

**Published:** 2009-07-20

**Authors:** Rebecca L Dunfee, Elaine R Thomas, Dana Gabuzda

**Affiliations:** 1 Department of Cancer Immunology and AIDS, Dana Farber Cancer Institute, Boston MA, USA; 2 Department of Pathology, Harvard Medical School, Boston MA, USA; 3 Department of Neurology, Harvard Medical School, Boston MA, USA

## Abstract

Macrophages in the central nervous system (CNS) and other tissues are an important cellular reservoir for human immunodeficiency virus type 1 (HIV) infection, particularly in the later stages of disease. Macrophage-tropic HIV strains have an enhanced capacity to enter cells expressing low levels of CD4 through mechanisms that are not well understood. Here, we use a panel of primary HIV envelopes from brain and lymphoid tissues to examine the relationship between neutralization sensitivity to reagents targeting the CD4 binding site and virus entry into macrophages. Neutralization assays using pseudotyped viruses showed an association between the capacity of HIV to enter macrophages and increased sensitivity to the broadly neutralizing monoclonal antibody (mAb) b12, which recognizes a conserved epitope overlapping the CD4 binding site, but not sensitivity to soluble CD4 (sCD4) or b6, a non-neutralizing CD4 binding site mAb. Furthermore, loss of an N-linked glycosylation site at position 386 in the V4 region of Env enhanced macrophage tropism together with b12 sensitivity, but not neutralization by sCD4, b6, or a broadly neutralizing AIDS patient serum. These findings suggest that exposure of the b12 epitope, rather than exposure of the CD4 binding site per se, enhances HIV macrophage tropism, possibly by exposing a region on the outer domain of gp120 that is initially recognized by CD4. These findings suggest overlap between specific gp120 determinants in or near the b12 epitope and those conferring macrophage tropism.

## Background

Human immunodeficiency virus type 1 (HIV) infects tissue macrophages, microglia, and other mononuclear phagocytes, which represent an important cellular reservoir for viral replication and persistence in brain and other macrophage-rich tissues (*i.e*., lung, gut, and bone marrow) [1-3]. HIV entry into cells is initiated by interaction between the envelope glycoprotein (Env) surface subunit gp120 and CD4, which induces a conformational change in gp120 that exposes the coreceptor binding site [4]. The interaction of CD4-bound gp120 with a coreceptor, usually CCR5 or CXCR4, triggers conformational changes in gp120 and the transmembrane subunit gp41 that enable fusion and virus entry. CCR5 is the primary coreceptor used for infection of macrophages [4-7]. CCR5 usage is neither necessary nor sufficient for macrophage tropism [8], however, suggesting that determinants other than those that specify coreceptor usage influence the capacity of HIV to replicate in macrophages.

Macrophages express lower levels of CD4 compared to CD4+ T-lymphocytes. Previous studies demonstrated that HIV macrophage tropism is associated with an enhanced capacity to use low levels of CD4 for fusion and entry [9-14]. We previously identified amino acid variants in the HIV Env that increase viral tropism for macrophages by enhancing gp120-CD4 affinity (N283 in the C2 region) or exposure of the CD4 binding site (loss of an N-linked glycosylation site at position 386 in the V4 region) [9,10]. However, HIV can also acquire an enhanced ability to enter macrophages by additional mechanisms that are not well defined.

The HIV envelope glycoproteins are the primary target for neutralizing antibodies *in vivo *[15,16]. The antibody response to acute HIV infection develops rapidly, and evolves concurrently with viral diversity during the course of disease, exerting strong selection pressure on viral evolution and leading to emergence of neutralization-resistant HIV variants [17,18]. The ability to generate neutralizing antibodies diminishes during disease progression, reflecting progressive loss of CD4 T-cell help and B-cell dysfunction.

HIV isolates that replicate efficiently in macrophages and microglia frequently exhibit increased sensitivity to neutralizing antibodies [11-13,19,20]. Consistent with these findings, a simian-human immunodeficiency virus (SHIV) isolated from infected rhesus macaques with neurological disease exhibited enhanced macrophage tropism together with increased sensitivity to neutralizing antibodies [21]. The HIV Env amino acid variant D386, which eliminates an N-linked glycosylation site and increases exposure of the conserved broadly neutralizing monoclonal antibody (mAb) b12 epitope overlapping the CD4 binding site, also enhances HIV macrophage tropism [10,22,23]. Previous studies reported that HIV macrophage tropism correlates with increased neutralization sensitivity to mAbs and other reagents that block Env-CD4 interactions but not with sensitivity to other entry inhibitors [22,23]. Collectively, these findings suggest that an association between enhanced HIV entry into macrophages and increased sensitivity to reagents targeting the CD4 binding site.

Here, we use a panel of viruses expressing primary HIV Envs from brain and lymphoid tissues [9,10,14] to further examine the association between neutralization sensitivity to reagents targeting the CD4 binding site and macrophage tropism. The capacity of HIV to enter macrophages correlated with neutralization sensitivity to the CD4 binding site mAb b12 and a broadly neutralizing HIV-infected patient serum, but not sensitivity to soluble CD4 (sCD4) or mAb b6, another mAb that targets the CD4 binding site. The loss of an N-linked glycosylation site at position 386 enhanced macrophage tropism together with sensitivity to mAb b12, but not sensitivity to sCD4, mAb b6, or HIV-infected patient serum. These findings suggest that exposure of the b12 epitope overlapping the CD4 binding site, rather than exposure of the CD4 binding site per se, enhances HIV macrophage tropism, possibly by exposing a region on the outer domain of gp120 that is initially recognized by CD4.

## Findings

We previously demonstrated that loss of an N-linked glycosylation site at position 386 in the V4 region of primary HIV Envs increases exposure of the b12 epitope and enhances macrophage tropism [10]. To better understand the relationship between macrophage tropism and sensitivity to reagents targeting the CD4 binding site, we used a panel of viruses containing CCR5-tropic (R5) primary HIV Envs cloned directly from brain and lymphoid tissues [9,10,14] to determine neutralization sensitivity to sCD4 and mAbs b12 and b6, which recognize neutralizing and non-neutralizing epitopes overlapping the CD4 binding site [24], respectively, and a broadly-neutralizing HIV-infected patient serum (Table 1). Env genes cloned into pCR3.1 from primary virus isolates or autopsy brain and lymphoid tissues from AIDS patients with HIV-associated dementia (HAD) were described previously [9,14,19]. HIV luciferase reporter viruses were generated by cotransfection of 293T cells with an HIV provirus with *env *deleted and *nef *replaced by luciferase (pNL4-3env^-^luc,) and pCR3.1-Env as described [19]. Cf2 cells [19] used as target cells for neutralization assays were cotransfected with pcDNA3-CD4 and pcDNA3-CCR5. HIV luciferase reporter viruses were incubated with a range of concentrations of human monoclonal Abs (mAbs), soluble CD4 (sCD4; Immunodiagnostics, Inc., Woburn, MA), or a broadly neutralizing HIV-1 serum (HIV-1 neutralizing serum (serum 2; [25]) obtained from L. Vujcic through the AIDS Research and Reference Reagent Program) 1 h prior to infection of Cf2 cells transiently expressing CD4 and CCR5. Cells were harvested 48 h post infection and assayed for luciferase activity.

**Table 1:** Neutralization sensitivity of primary HIV-1 Envs with variable macrophage tropism to gp120 mAbs, soluble CD4, and a broadly neutralizing HIV-infected patient serum

Patient	Tissue^a^	Env clone	MDM entry^b^	b12 IC_50_^c^	b6 IC_50_^c^	sCD4 IC_50_^c^	PS IC_50_^c^
MACS2	FL	8–12	35478	3.37	> 20	1.03	80.9
		9–15	1765	1.15	> 20	0.22	76.4
	LN	10–15	13001	0.99	> 20	> 20	< 50
	SP	6–18	59224	10.89	> 20	> 20	< 50
MACS3	FL	12–27	13810	> 20	1.87	1.10	< 50
		5	3402	> 20	6.78	0.40	< 50
	LN	2	1957	> 20	> 20	3.22	< 50
		20	16658	> 20	> 20	> 20	< 50
UK1	FL	2–13b	4378	0.05	> 20	5.44	< 50
	SP	6–20	812689	0.03	> 20	2.39	124.2
		20	35723	0.03	> 20	0.27	230.2
UK7	FL	6–24	9659	5.53	> 20	7.68	145
		1–4	13207	11.37	> 20	> 20	128
	isolate	br34	496588	0.26	> 20	0.45	332.2
	LN	7–6	5260	5.55	> 20	> 20	< 50
controls		YU2	38052	5.47	> 20	1.14	72.4
		YU2 N386D	114755	2.79	> 20	0.45	115.2
		JRFL	215305	0.18	> 20	3.72	107.3
		JRFL N386D	320642	0.09	> 20	6.05	92.7

^a ^FL, frontal lobe (brain); LN, lymph node; SP, spleen

^b ^Luciferase activity in monocyte-derived macrophages (MDM) infected with pseudotyped luciferase-expressing reporter viruses [10,14].

^c ^mAb (μg/ml), soluble CD4 (sCD4; μg/ml), or HIV-infected patient serum (PS; reciprocal serum dilution) concentration at which luciferase expression was reduced by 50% compared to infection in the absence of mAb (IC_50_).

**Figure 1:**
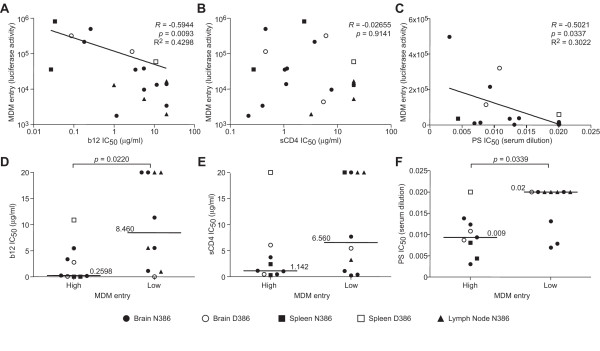
**Enhanced HIV entry into macrophages is associated with sensitivity to neutralizing mAb b12 and a broadly neutralizing HIV-infected patient serum**. HIV luciferase reporter viruses pseudotyped with primary HIV Envs cloned directly from brain, spleen, or lymph node tissues from AIDS patients with HAD were incubated with a range of concentrations of human mAb b12 (A and D), soluble CD4 (sCD4; B and E), or HIV-1 neutralizing patient serum (PS; C and F) 1 h prior to infection of Cf2 cells transiently expressing CD4 and CCR5. Cells were harvested 48 h post infection and assayed for luciferase activity. (A, B, C) The concentrations at which luciferase expression was reduced by 50% compared to infection in the absence of mAb (IC_50_) were calculated and plotted as a function of MDM entry [10,14]. R and p values were determined by Spearman correlation. (D, E, F) b12, sCD4, and PS IC50s of HIV Envs with low to intermediate MDM infectivity (< median; median = 16,658 relative luciferase units) were compared to Envs with intermediate to high MDM infectivity (> median). Monocyte-derived macrophages (MDM) were isolated from peripheral blood mononuclear cells from healthy HIV-1-negative donors by plastic adherence and cultured in RPMI 1640 medium supplemented with 10% FBS, and 10 ng/ml macrophage colony stimulating factor (M-CSF) [8]. The MDM entry and sequence data were reported previously [10,14]. Env clones containing either the N386 or D386 variant are indicated by closed and open symbols, respectively. MDM were prepared as above in 48-well plates and infected with 2 × 10^4 ^^3^H cpm RT units of Env pseudotyped virus stock. Cells were lysed 6 days post-infection and assayed for luciferase activity. Significant differences between groups (p < 0.05, Mann-Whitney test) are indicated by a *.

Viruses pseudotyped with HIV Envs that mediate high levels of entry into macrophages had increased sensitivity to mAb b12 and the HIV-infected patient serum compared to viruses expressing Envs that mediate low levels of entry in macrophages (Figure 1A, C, D, F; A and 1C, R = -0.5944, p = 0.0093 and R = -0.5021, p = 0.034, respectively, Spearman correlation; 1D and 1F, p = 0.022 and 0.034, respectively, Mann-Whitney test). In contrast, sensitivity to sCD4 or the non-neutralizing mAb b6 did not correlate with levels of HIV entry into macrophages (Figure 1B, E, p = 0.9141; and data not shown). Furthermore, there was no correlation between neutralization sensitivity to mAb b12 and neutralization sensitivity to sCD4 (p = 0.3279; Additional file 1). These results suggest that macrophage tropism is associated with increased sensitivity to mAb b12, but not sensitivity to sCD4 or the non-neutralizing CD4 binding site mAb b6. Thus, macrophage tropism was associated with increased exposure of the b12 epitope overlapping the CD4 binding site, rather than exposure of the CD4 binding site per se.

**Figure 2:**
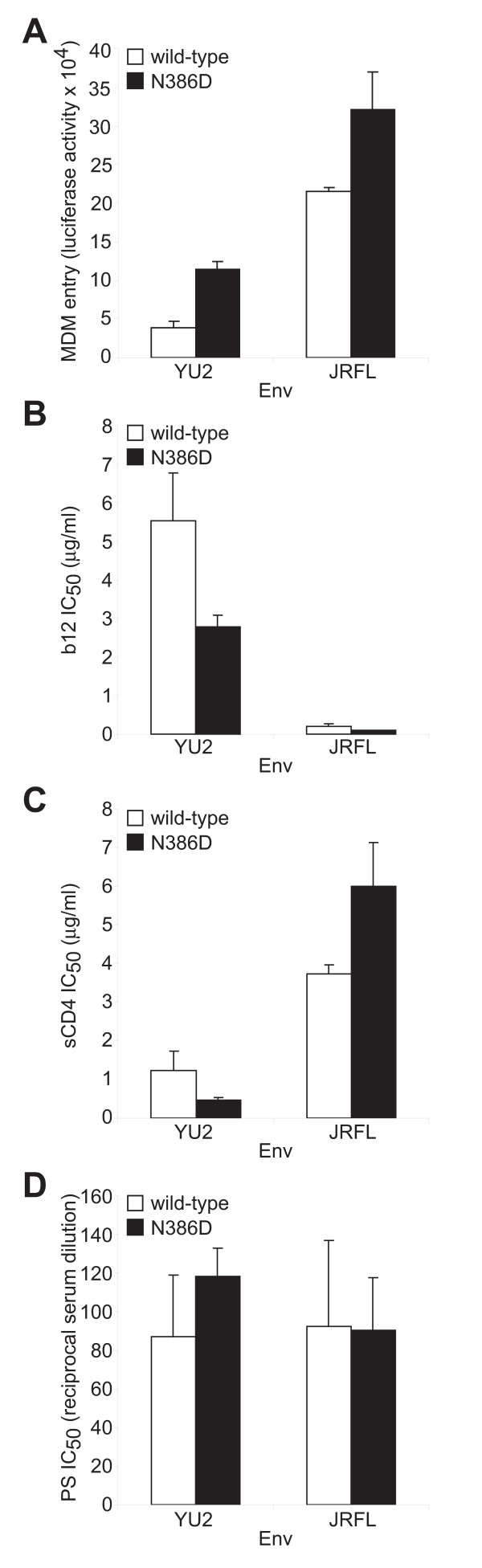
**Loss of an N-linked glycan at position 386 in primary HIV Envs enhances macrophage tropism and neutralization sensitivity to mAb b12**. (A) MDM were infected with luciferase-expressing reporter viruses expressing wild-type or N386D mutant Envs. Cells were lysed 6 days post-infection and analyzed for luciferase activity. (B, C, D) Luciferase-expressing reporter viruses expressing wild-type or N386D mutant Envs were incubated with a range of concentrations of human mAb b12 (B), sCD4 (C), or a HIV-1 neutralizing patient serum (PS; D) 1 h prior to infection of Cf2 cells transiently expressing CD4 and CCR5. Cells were harvested 48 h post infection and assayed for luciferase activity. Data are expressed as the concentrations at which luciferase expression was reduced by 50% compared to infection in the absence of mAb (IC_50_). Error bars represent standard deviations.

Elimination of an N-linked glycan at position 386 in the macrophage-tropic primary HIV Envs YU2 and JRFL enhances entry into macrophages by 200% and 49%, respectively (Table 1, Figure 2A and [10]). To determine whether removal of the N-linked glycan at position 386 also influences sensitivity to b12 or other reagents that target the CD4 binding site, we investigated neutralization of viruses expressing YU2 and JRFL wild-type and N386D mutant Envs with mAbs b12 and b6, sCD4 and the broadly neutralizing HIV-infected patient serum. The N386D change in both YU2 and JRFL resulted in a 2-fold increase in sensitivity to neutralization by mAb b12 compared to that of the wild-type parental Envs (Table 1 and Figure 2B). The N386D change in YU2 resulted in increased sensitivity to sCD4, whereas the N386D change in JRFL decreased sCD4 sensitivity compared to the wild-type parental Envs (Table 1 and Figure 2C). YU2 and JRFL wild-type and N386D mutant viruses had similar sensitivities to neutralization by mAb b6 and the HIV-infected patient serum (Table 1, Figure 2D, and data not shown). These results are consistent with our previous study in which the reverse mutation D386N, which restored the N-linked glycan at position 386 in the macrophage-tropic UK1br and Macs2br13 Envs, decreased sensitivity to neutralization by mAb b12 along with replication in macrophages [10].

Our findings demonstrate an association between the capacity of HIV to enter macrophages (i.e., macrophage tropism) and neutralization sensitivity to the CD4 binding site mAb b12, but not sensitivity to the non-neutralizing mAb b6 or sCD4. Furthermore, we show that loss of an N-linked glycosylation site at position 386 in the macrophage-tropic HIV YU2 and JRFL Envs enhances macrophage tropism along with neutralization sensitivity to b12, but not neutralization sensitivity to sCD4, b6, or a broadly neutralizing AIDS patient serum. These findings suggest overlap between specific gp120 determinants in or near the b12 epitope and those conferring macrophage tropism.

CD4, b12, and b6 have overlapping binding sites on gp120 [24,26]. The b12 mAb recognizes a conserved epitope on the neutralizing face of gp120 overlapping the CD4 binding site, while b6 recognizes a different epitope that partially overlaps the binding sites for b12 and CD4 [24]. The initial Env-CD4 interaction readily dissociates, and conformational changes in Env induced by CD4 binding increase the stability of the Env-CD4 complex before subsequent structural rearrangements allow coreceptor binding [26,27]. b12 contact occurs at the exposed surface on the outer domain of gp120 that is initially recognized by CD4 [26]. Furthermore, b12 is the only antibody that targets the CD4 binding site and also recognizes Env in the CD4-bound, stabilized conformation adopted before coreceptor binding [26]. These observations support the idea that exposure of the b12 epitope enhances HIV entry into macrophages, which express low levels of CD4 compared to T-cells, possibly by exposing a region on the outer domain of gp120 initially recognized by CD4.

The loss of a glycosylation site at HIV Env position 386 increases exposure of the b12 epitope [10,22,28], probably due to loss of steric hindrance, and also enhances macrophage tropism in a strain-dependent manner [10]. Loss of a glycosylation site at 386 does not predict b12 sensitivity [10,22,28], however, suggesting that other Env determinants influence exposure of the b12 epitope. Duenas-Decamp et al. showed that an arginine at position 373 in the C3 region, proximal to the CD4 binding site, increased resistance to b12 neutralization [22]. However, the HIV Envs in the present study all have methionine or threonine at position 373, and b12 neutralization did not correlate with amino acid differences at this position (data not shown). Thus, the influence of amino acid changes at position 373 on exposure of the b12 epitope is highly strain-dependent.

We found that a majority of macrophage-tropic HIV Envs are sensitive to b12 neutralization. A recent study demonstrated that early transmitted HIV variants replicate in T-cells but have relatively low capacity to replication in MDM [29]. HIV viruses isolated from late-stage AIDS patients have enhanced macrophage tropism together with increased neutralization sensitivity to b12 compared to viruses isolated from asymptomatic HIV-infected individuals [30]. Anti-CD4 binding site antibodies are generated after HIV infection, and can be detected in most HIV-infected individuals [31-33]. Broader and more potent neutralizing antibody responses are often due to the presence of neutralizing antibodies targeting the CD4 binding site [31-33]. However, only rare individuals develop broadly neutralizing anti-CD4 binding site antibodies, so b12-like antibodies are uncommon in HIV-infected patients. The neutralizing activity of sera from long-term nonprogressors is partially attributable to the presence of antibodies that target the CD4 binding site [32,34], suggesting these antibodies may play a role in controlling viral replication *in vivo*. Neutralizing antibody responses that target the b12 binding site may exert negative selection pressure against macrophage-tropic HIV variants until the later stages of HIV disease. The brain may be a site for emergence and persistence of neutralization-sensitive macrophage-tropic HIV variants, particularly in the setting of a weak humoral immune response [35,36].

In conclusion, macrophage-tropic HIV strains play a role in the establishing long-lived cellular reservoirs in macrophage-rich tissues that include brain, lung, gut, and bone marrow [1-3]. Furthermore, macrophages are the principal source of virus after CD4+ T cells are depleted [37]. In this study, we demonstrate an association between HIV macrophage tropism and neutralization sensitivity to the CD4 binding site mAb b12. In contrast, there was no association with neutralization sensitivity to the non-neutralizing CD4 binding site mAb b6 or sCD4. These findings suggest overlap between specific gp120 determinants that increase exposure of the b12 epitope and those conferring macrophage tropism. Future studies will be important to better understand immune selection pressures that drive HIV evolution towards variants with enhanced macrophage tropism, and the role of neutralizing antibodies that target the CD4 binding site in these processes.

## Competing interests

The authors declare that they have no competing interests.

## Authors' contributions

R.L.D. and D.G. designed research; R.L.D. and E.R.T performed research; R.L.D. and D.G. analyzed data; and R.L.D. and D.G. wrote the paper.

## Supplementary Material

Additional file 1 **Supplementary Figure. HIV Env neutralization sensitivity to mAb b12 does not correlate neutralization sensitivity to sCD4**. HIV luciferase reporter viruses were incubated with a range of concentrations of human mAb b12 or sCD4 1 h prior to infection of Cf2 cells transiently expressing CD4 and CCR5. Cells were harvested 48 h post infection and assayed for luciferase activity. Data are expressed as the concentrations at which luciferase expression was reduced by 50% compared to infection in the absence of mAb or sCD4 (IC_50_). (A) sCD4 IC_50_s were plotted as a function of b12 IC_50_s. R and p values, Spearman correlation. (B) sCD4 IC_50_s of HIV Envs with low to intermediate b12 sensitivity (< median; median = 3.374 μg/ml) were compared to Envs with intermediate to high b12 sensitivity (> median). p values, Mann-Whitney test. Click here for file
