# Supplementary figures and images for: Enhanced macrophage tropism of HIV in brain and lymphoid tissues is associated with sensitivity to the broadly neutralizing CD4 binding site antibody b12
Source: Retrovirology. 2009 Jul 20;6:69. doi: 10.1186/1742-4690-6-69 (PMC2717910; doi:10.1186/1742-4690-6-69)

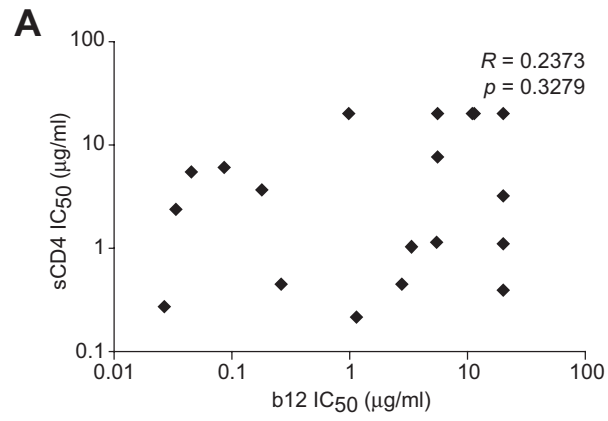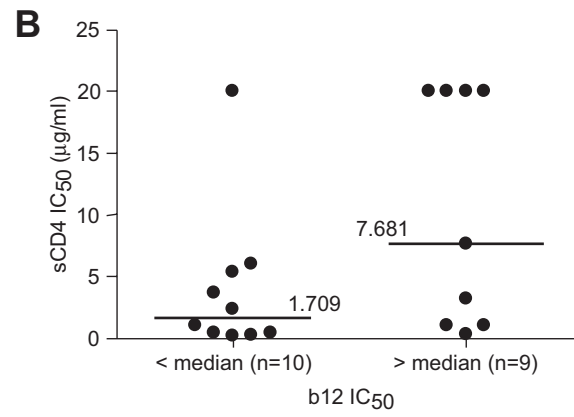

Supplement: Additional file 1 — Supplementary Figure. HIV Env neutralization sensitivity to mAb b12 does not correlate neutralization sensitivity to sCD4. HIV luciferase reporter viruses were incubated with a range of concentrations of human mAb b12 or sCD4 1 h prior to infection of Cf2 cells transiently expressing CD4 and CCR5. Cells were harvested 48 h post infection and assayed for luciferase activity. Data are expressed as the concentrations at which luciferase expression was reduced by 50% compared to infection in the absence of mAb or sCD4 (IC50). (A) sCD4 IC50s were plotted as a function of b12 IC50s. R and p values, Spearman correlation. (B) sCD4 IC50s of HIV Envs with low to intermediate b12 sensitivity (< median; median = 3.374 μg/ml) were compared to Envs with intermediate to high b12 sensitivity (> median). p values, Mann-Whitney test. [file 1742-4690-6-69-S1.pdf]
